# Draft Genome Sequence of *Streptomyces* sp. Strain BR123, Endowed with Broad-Spectrum Antimicrobial Potential

**DOI:** 10.1128/MRA.00972-20

**Published:** 2020-10-08

**Authors:** Neelma Ashraf, Andreas Bechthold, Munir Ahmad Anwar, Shazia Khaliq

**Affiliations:** a Industrial Biotechnology Division, National Institute for Biotechnology and Genetic Engineering (NIBGE), Constituent College, Pakistan Institute of Engineering and Applied Sciences (PIEAS), Faisalabad, Pakistan; b Department of Pharmaceutical Biology and Biotechnology, Institute of Pharmaceutical Sciences, University of Freiburg, Freiburg im Breisgau, Germany; Queens College

## Abstract

The genome of *Streptomyces* sp. strain BR123, isolated from rhizospheric soil that exhibited promising antimicrobial properties, was sequenced and assembled. Here, we report an 8,157,040-bp genome sequence with a G+C content of 72.63%. This genome sequence enlightens the genes responsible for the production of secondary metabolites and antimicrobial compounds by this strain.

## ANNOUNCEMENT

*Streptomyces* members are aerobic, Gram-positive filamentous bacteria belonging to the order *Actinomycetales* of *Actinobacteria*. Bacteria in this genus are well reported for their production of bioactive secondary metabolites that have great biofunctional diversity and different applications, such as antibacterial, antifungal, antiviral, anticancer, immunosuppressant, insecticidal, and herbicidal, which make them suitable for use as pharmaceuticals and in agricultural industries (1–4). More than 7,630 bioactive secondary metabolites have been reported to be produced only by *Streptomyces* spp. (5, 6).

*Streptomyces* sp. strain BR123 was isolated from rhizospheric soil surrounding the roots of a Helianthus annuus plant and was grown for 7 days at 28°C on casein-starch-peptone-yeast extract-malt extract (CSPY-ME) medium (7). A single colony was inoculated in CSPY-ME broth and incubated at 30°C for 72 h. Genomic DNA was extracted through a bead method (8). Culture containing three beads in an Eppendorf tube was washed with extraction buffer endowed with lysozyme and was allowed to incubate at 37°C for 25 min. For protein and RNA denaturation and removal, proteinase K and RNase A were added, followed by incubation for 5 min at 65°C. Purification of extracted DNA was performed by resuspending it in elution buffer (EB) with an equal volume of solid-phase reversible immobilization (SPRI) beads. Quantification of DNA was performed in triplicates in an Eppendorf AF2200 plate reader with the Quant-iT double-stranded DNA (dsDNA) high-sensitivity (HS) assay. The Nextera XT library preparation kit (Illumina, San Diego, CA, USA) was employed for genomic DNA library preparation following the manufacturer’s protocol with some modifications, as follows: for PCR, 2 ng of DNA instead of 1 ng was used as input, and elongation time was exceeded from 30 seconds to 1 min. The Hamilton Microlab STAR automated liquid-handling system was employed for DNA quantification and library preparation. By using a Roche light cycler 96 quantitative PCR (qPCR) machine, pooled Illumina libraries were generated and quantified with the Kapa Biosystems library quantification kit. The Illumina HiSeq platform was used for base calling by the 250-bp paired-end protocol. For all specified software, default parameters were used.

Paired-end reads yielding 30× coverage were checked for quality control, adapted reads were trimmed by using Trimmomatic 0.30 with a sliding window quality cutoff of Q15 (9), and quality was evaluated by means of the house scripts C program for matching variants in combination with three software packages, i.e., SAMtools 1.9 (https://sourceforge.net/projects/samtools/files/samtools/1.9/), BEDtools 2.28 (https://bedtools.readthedocs.io/en/latest/), and BWA-MEM 7.12 (10). By the use of SPAdes version 3.7, *de novo* assembly was performed (11), and annotation of contigs was done by using Prokka 1.11 (12). The NCBI Prokaryotic Genome Annotation Pipeline (PGAP) version 4.12 (https://www.ncbi.nlm.nih.gov/genome/annotation_prok/) was used to assemble metrics.

The genome assembly yielded 723 contigs with an *N*_50_ value of 22,797 bp, and the largest contig has 109,551 bp. The genome of *Streptomyces* sp. strain BR123 contains 8,157,040 bp with a G+C content of 72.63%, a value highly similar to those reported in *Streptomyces* spp. (13, 14). There are 7,070 coding sequences with 70 tRNAs and 3 noncoding RNAs (ncRNAs).

### Data availability.

This whole-genome shotgun project has been deposited at GenBank under whole-genome sequencing project number JACBGN000000000, BioProject number PRJNA643667, SRA number SRR12527047, and BioSample accession number SAMN15423153. The 16S rRNA gene sequence has been deposited at DDBJ/ENA/GenBank under the accession number MT799988.
